# AI/ML-based strategies for enhancing equity, diversity, and inclusion in randomized clinical trials

**DOI:** 10.1186/s13063-026-09537-2

**Published:** 2026-02-13

**Authors:** Shashidar Reddy Abbidi, Debashree Sinha

**Affiliations:** Charlotte, NC USA

**Keywords:** Clinical trials, Artificial intelligence, Machine learning, Fairness, Health equity, Diversity, Inclusion, Bias detection, Recruitment optimization

## Abstract

This paper introduces a conceptual framework designed to embed equity, diversity, and inclusion (EDI) across all stages of the clinical trial lifecycle. Randomized clinical trials (RCTs) remain the most reliable method for evaluating medical treatments, yet persistent gaps in representation undermine their validity and fairness. Women, older adults, racial and ethnic minorities, and socioeconomically disadvantaged groups are often underrepresented, raising concerns about whether trial results can be generalized to all patients. This lack of inclusivity not only limits scientific rigor but also risks reinforcing existing health disparities. Recent advances in artificial intelligence (AI) and machine learning (ML) provide new opportunities to address these challenges. These technologies can support more inclusive study designs, enable targeted recruitment of underrepresented populations, and monitor diversity in real time throughout the trial process. They can also be applied to analyze outcomes with fairness-aware methods, helping ensure that results are meaningful across diverse subgroups. In this work, we propose an AI/ML-based framework aimed at operationalizing equity, diversity, and inclusion in clinical research. The framework integrates predictive modeling, adaptive trial designs, and continuous bias detection with ethical and legal safeguards to ensure responsible deployment. By embedding fairness into every stage of the trial lifecycle, this approach offers a pathway toward more representative and trustworthy evidence in medical science. Our analysis reveals the persistent gaps across demographic groups in current RCTs, demonstrating the urgent requirement for systematic intervention. This study also contributes a comprehensive AI/ML framework that operationalizes equity through predictive modeling, adaptive designs, and continuous bias monitoring, providing a structured pathway for researchers to enhance both the scientific validity and ethical integrity of clinical trials.

## Introduction

Randomized clinical trials (RCTs) have long been looked as the gold standard in medical research, offering the most reliable evidence for assessing the safety, efficacy, and overall effectiveness of new drugs, therapies, and medical devices. The strength of RCTs lies in their structured method and the principle of randomization, which reduces bias and ensures that outcomes can be attributed to the intervention under study rather than external factors. For this reason, RCTs serve as the benchmark against which other types of clinical evidence are measured [1].

However, the validity of these trials is increasingly challenged when the participants enrolled fail to reflect the diversity of the real-world populations that the treatments are ultimately intended to serve. Over the past decade, numerous studies have documented consistent inequities in trial participation. Women, elderly populations, ethnic and racial minorities, and individuals from low-income backgrounds are frequently underrepresented across multiple therapeutic domains [2]. For instance, cardiovascular trials have historically enrolled disproportionately fewer women, despite heart disease affecting men and women at nearly equal rates. Likewise, oncology trials often exclude older adults, leaving critical gaps in evidence for those most vulnerable to cancer. Such disparities are not simply academic; they carry profound clinical consequences. When trial populations lack diversity, the evidence-based treatments may not be equally effective or accessible for marginalized communities, thereby perpetuating existing health inequities.

Recent advances in AI and ML offer promising avenues to confront these challenges. Unlike conventional trial methodologies that rely heavily on static protocols and clinician judgment, AI/ML systems can process vast and heterogeneous datasets, including electronic health records, genomic data, patient registries, and real-world health evidence. These technologies are uniquely positioned to detect hidden biases, forecast recruitment shortfalls, and recommend corrective measures in real time. For example, predictive models can simulate how interventions may perform across underrepresented subgroups or flag imbalances in recruitment during trial enrollment phases. At the same time, AI-powered tools can help expand outreach by identifying underserved populations and customizing communication strategies to reduce cultural or socioeconomic barriers to participation.

Importantly, the role of AI/ML extends beyond recruitment. Fairness-aware algorithms can be embedded across the entire trial lifecycle from design and monitoring to outcome analysis, ensuring that equity remains a guiding principle rather than a retrospective concern. Methods such as stratified randomization can be strengthened through machine learning, while bias detection models can identify disparities in trial results across demographic subgroups. Collectively, these approaches mark a shift toward clinical research that is more inclusive, trustworthy, and socially responsible [3].

Guided by this vision, the present work introduces a conceptual framework that leverages AI/ML to enhance equity, diversity, and inclusion (EDI) in randomized clinical trials. The framework integrates key components such as fairness metrics, synthetic data generation, recruitment optimization, and ongoing bias monitoring into a unified pipeline designed to be adaptive and scalable. By situating AI/ML at the intersection of scientific rigor and ethical responsibility, this paper aims to demonstrate how emerging technologies can strengthen both the validity of clinical evidence and the commitment to equitable healthcare. Guzi et al. proposed a framework for patient data management, but in our framework, we proposed a technique using artificial intelligence, which is the need of the current time to make predictions, making our framework advanced [4].

This research is guided by two main research objectives. First, we aim to thoroughly identify and quantify the specific obstructions and biases that contribute to underrepresentation in clinical trials, inspecting how these discrepancies vary across therapeutic areas and demographic groups. Second, we want to develop and validate a comprehensive AI/ML-driven framework that can proactively address these inequities throughout the trial lifecycle, from initial design through final analysis. While previous literature has documented gaps in trial representation, this work advances the field by proposing a comprehensive, fairness-aware conceptual framework designed to bridge these gaps.

### Novel contributions and advances beyond existing work

This paper introduces a closed loop process where outcomes directly contribute to future trial designs, representing a major shift from current recruitment strategies to data-driven, advanced, and adaptive equity management.

## EDI challenges in clinical trials

While randomized clinical trials (RCTs) are the benchmark for medical research, they struggle with a lack of diversity in participant enrollment. Several systemic issues create this problem. First, strict eligibility criteria, meant to ensure safety, often exclude older adults, pregnant women, and people with multiple health conditions, making the trial population younger and healthier than the general public.

Socioeconomic and geographic barriers also play a role. Trial sites are often in urban areas, which limits access for people in rural or low-income regions. The high costs of transportation, time off work, and childcare can be a significant burden that financial compensation from sponsors often does not cover [5].

Recruitment methods further contribute to the issue. They tend to favor patients already connected to large medical centers, overlooking communities with limited healthcare access or a historical mistrust of medical institutions, such as racial and ethnic minorities. Cultural and linguistic barriers also exist, as recruitment materials are often not available in different languages or tailored to various cultural beliefs, excluding many potential participants.

This lack of diversity has major consequences. It weakens the generalizability of trial results and risks worsening healthcare disparities by creating evidence that benefits well-represented groups while ignoring the needs of marginalized communities. This also erodes public trust in medical research.

To address these challenges, a two-pronged approach is needed. Policy changes are required to enforce inclusion standards, while new technologies like AI and ML can provide practical tools to identify and correct biases in real time. By combining these strategies, clinical research can become both more scientifically sound and socially responsible. Unlike traditional trial methods that rely on protocols and judgement, AI/ML methods depend on the dataset value and training the dataset to create a model which can provide much better accuracy as compared to traditional methods using human intelligence.

## AI/ML strategies for promoting EDI

### Trial design

AI-driven synthetic data generation allows simulation of diverse cohorts, while predictive modeling helps assess the inclusivity of eligibility criteria [4]. Reinforcement learning has been applied to adaptively redesign protocols that account for underrepresented populations.

### Recruitment

Machine learning applied to EHRs supports identification of eligible participants from minority groups. Fairness-aware recommender systems can suggest optimal recruitment sites, as demonstrated by the FRAMM (Fair Ranking with Missing Modalities) framework, which improved minority enrollment by up to 60% in simulation [6].

### Enrollment and monitoring

Fairness-aware eligibility optimization can flag exclusionary criteria, while AI-based telehealth platforms improve participation of rural and mobility-limited patients [7]. Bias detection algorithms monitor enrollment diversity in real time.

### Analysis and outcomes

Explainable AI methods allow subgroup-specific analysis of treatment effects, while fairness metrics such as demographic parity and equalized odds validate equitable outcomes [8]. ML clustering can also uncover hidden disparities in treatment responses.

## Machine learning in clinical trial risk assessment

**Fig. 1:**
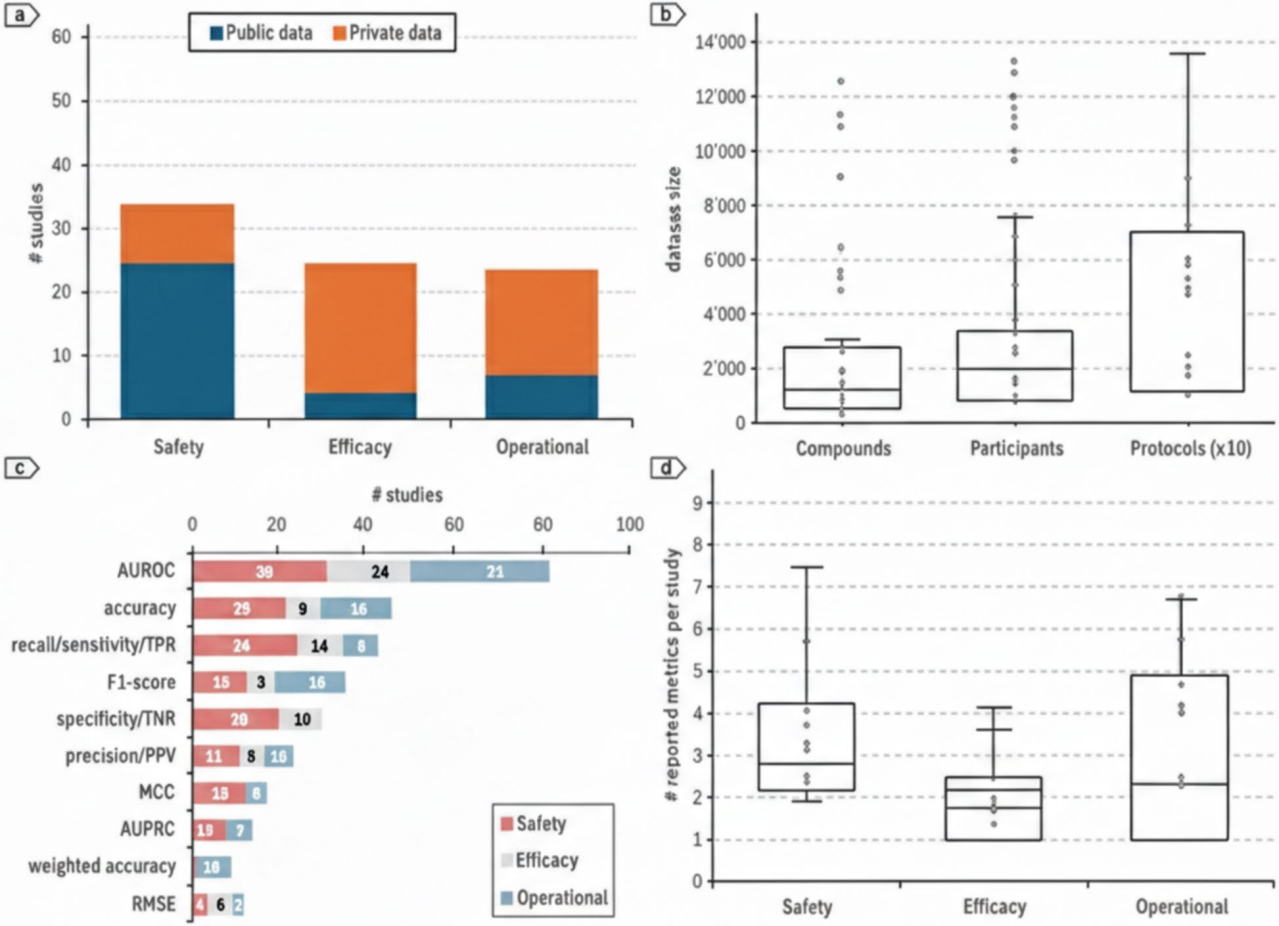
Overview of machine learning applications in clinical trial risk evaluation. **a** Distribution of public and private datasets used across safety, efficacy, and operational studies. **b** Dataset sizes reported for compounds, participants, and trial protocols. **c** Performance metrics are most frequently applied in published risk assessment studies. **d** Number of evaluation metrics reported per study across different trial domains

To better understand how artificial intelligence and machine learning approaches are currently applied within clinical research, we analyzed recent studies that utilized ML for risk evaluation in clinical trials. It should be noted that figures and performance trends presented here synthesize current literature illustratively, not through systematic quantitative analysis. AI/ML efficacy hinges on high-quality data and bias mitigation. Figure 1 provides a consolidated view of the key trends across safety, efficacy, and operational domains.

Sub-figure (a) highlights the balance between public and private datasets in different trial contexts. Safety-related studies show a strong reliance on publicly available datasets, reflecting the availability of adverse event reporting systems and pharmacovigilance databases. In contrast, efficacy studies rely more heavily on proprietary datasets, often derived from pharmaceutical pipelines and trial sponsors. Operational studies, such as those focused on recruitment efficiency or site performance, demonstrate a mixed use of both public and private sources.

Sub-figure (b) presents the distribution of dataset sizes, covering compounds, participants, and trial protocols. While datasets on compounds and participants tend to remain relatively modest in scale, protocol-based datasets show far greater variability, with some studies involving extremely large samples. This suggests that operational risk modeling often requires more complex and voluminous data integration compared to other domains.

Sub-figure (c) examines the performance metrics most frequently reported in risk assessment studies. Common measures include AUROC, accuracy, recall, and F1-score, reflecting a strong emphasis on predictive power and classification balance. However, metrics such as MCC, AUPRC, and RMSE are less frequently employed, suggesting that many studies prioritize broad performance measures while overlooking finer aspects of model reliability and calibration.

Finally, sub-figure (d) compares the number of evaluation metrics reported per study. Safety-focused studies tend to report a wider range of metrics, possibly due to the higher regulatory scrutiny and ethical importance of adverse event prediction. In contrast, efficacy and operational studies often limit their reporting to one or two standard measures, raising questions about consistency and transparency in ML-based trial risk assessment.

Overall, the figure demonstrates that while ML methods are gaining traction in clinical trial risk evaluation, there are still gaps in standardization, dataset accessibility, and the comprehensiveness of performance reporting. These findings reinforce the need for fairness-aware, standardized, and transparent AI frameworks that not only optimize predictive accuracy but also ensure inclusiveness and equity in trial outcomes.

## Conceptual framework: a fairness-aware AI pipeline

We propose an AI-enabled trial framework consisting of five integrated components, designed to enhance EDI across the RCT lifecycle. Standardizing demographic and diversity variables using clinical data standards like CDISC SDTM and ADaM ensures consistent applicability of fairness metrics across sponsors and therapeutic areas.

### Pre-trial data audit

Systematic auditing of available datasets (EHRs, registries, observational data) to quantify demographic representation. Fairness metrics such as representation ratios and subgroup balance are computed [8]. AI tools identify systemic gaps.

### Fairness-aware trial design

Synthetic data generation simulates underrepresented groups, enabling feasibility testing. Reinforcement learning can optimize adaptive trial designs, balancing scientific rigor with diversity.

### AI-driven recruitment optimization

Predictive models applied to EHRs identify eligible participants. The FRAMM algorithm [9] exemplifies fair site selection. Tools include multilingual chatbots, geo-analytics models, and recommender systems for outreach.

### Continuous bias monitoring

Fairness-aware monitoring tools track recruitment diversity in real time. Disparities are flagged automatically, enabling mid-trial interventions. Federated learning approaches enhance monitoring while preserving privacy [10]. To address practical CDM concerns, this real-time monitoring framework accounts for inherent data latency from site entry into EDC systems by utilizing buffered data streams for analysis.

### Equitable outcome validation

AI models trained with fairness constraints ensure consistent predictive performance across groups. Explainable AI techniques provide transparency regarding treatment effects. Fairness metrics validate equity of results [11].

### Feedback loop

The framework is designed as a closed-loop system: outcome analysis feeds back into design and recruitment strategies for future trials.

**Fig. 2:**
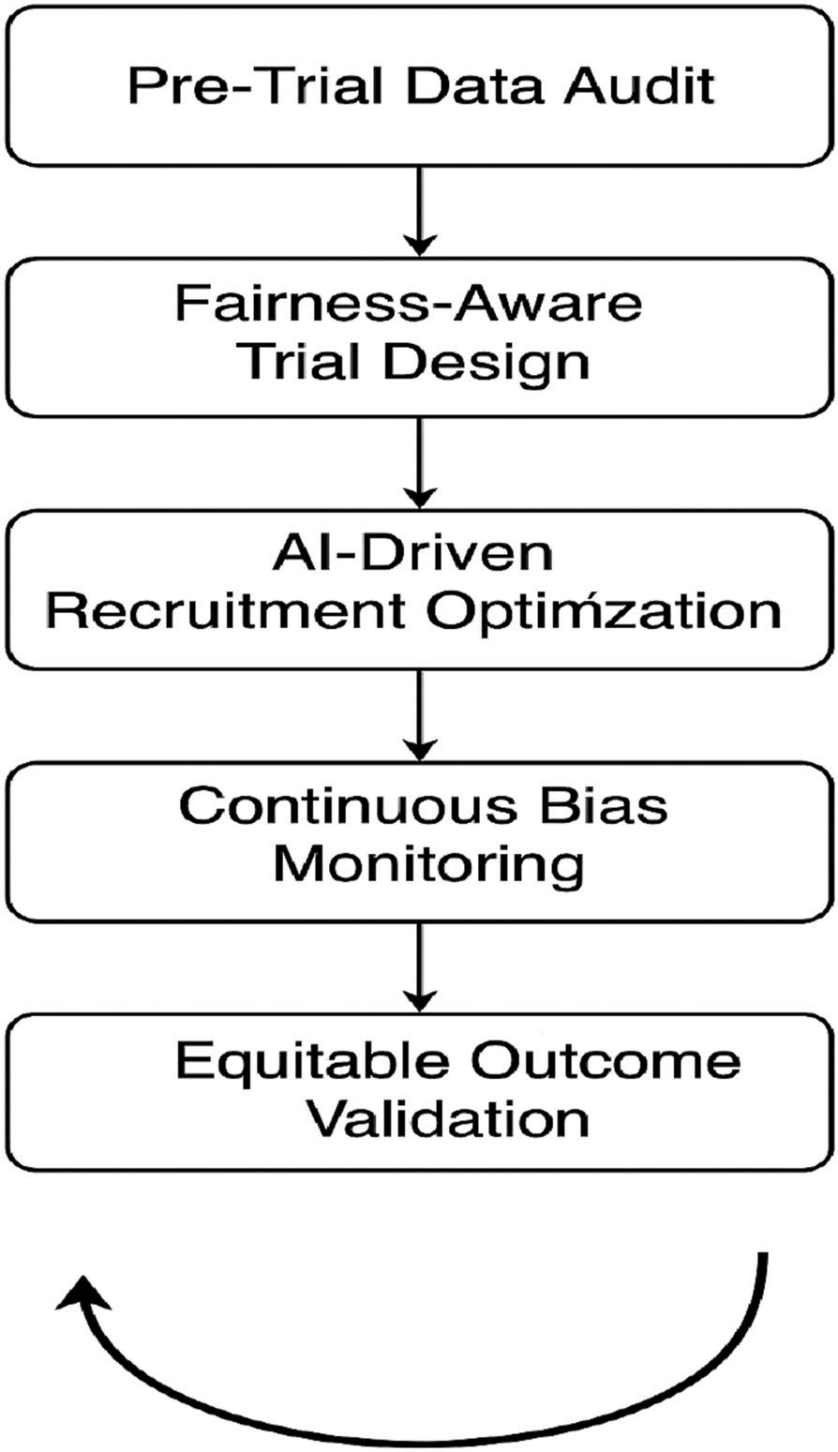
The proposed framework for enhancing equity, diversity, and inclusion in RCT

The proposed framework introduces an *AI/ML-driven pipeline* designed to strengthen equity, diversity, and inclusion (EDI) throughout the lifecycle of randomized clinical trials (RCTs). The framework operates through a structured sequence of stages, as illustrated in Fig. 2, and emphasizes fairness-aware intervention at every critical phase, beginning from trial design to outcome validation.

The first stage, *pre-trial data audit*, ensures that the datasets used for planning trials are examined for potential biases. By applying fairness metrics such as demographic parity and subgroup balance analysis, underrepresented populations can be identified prior to recruitment. The second stage, *fairness-aware trial design*, incorporates stratified randomization and fairness constraints to ensure proportionate representation across gender, ethnicity, and age groups. Where necessary, synthetic data generation techniques, such as GANs or SMOTE, are employed to counter the lack of minority subgroup data and to simulate equitable recruitment scenarios. The next step is AI-driven recruitment optimization; this uses smart technology to find and engage with people who are often left out of studies. By analyzing information from health records and using tools to understand language, it helps customize outreach so it is more effective for different groups. The goal is to make sure marginalized communities are actively involved from the start. Next is continuous bias monitoring, which uses real-time dashboards and special algorithms to keep an eye on how diverse the participant group is as the trial unfolds. If the system detects a lack of representation, it can automatically suggest changes, like adjusting recruitment goals, to keep the study on track for fairness.

The final component is equitable outcome validation. Here, the trial results are carefully checked to see how different demographic groups were affected. By using advanced analysis tools, researchers can find hidden biases and make sure the results are fair and applicable to everyone, not just a select group. What makes this framework truly special is that it is designed as a continuous loop. The insights gained from analyzing the outcomes are fed back into the beginning of the process, the pre-trial data audit. This creates a cycle of improvement, so each new trial can build on the last one to be more fair and inclusive. The visual diagram of the framework shows this progression and the feedback loop, highlighting its adaptive and cyclical nature. It is a comprehensive, scalable approach to making clinical trials scientifically sound and ethically responsible.

## Ethical, legal, and social considerations

The growing use of AI in the clinical trials offers exciting opportunities, but it also introduces a set of ethical, legal, and social challenges that must be carefully navigated. While these technologies have the potential to improve equity, diversity, and inclusion (EDI), they can also deepen existing inequalities if they are not designed and applied responsibly.

A central concern is *algorithmic bias*. ML models trained on datasets that are incomplete, imbalanced, or historically skewed can unintentionally reinforce patterns of exclusion. For example, if the electronic health records used to build a recruitment algorithm underrepresent minority groups, the model may continue to deprioritize those communities rather than helping to include them. Addressing this risk requires deliberate safeguards, such as the use of fairness metrics, systematic bias audits, and careful selection of training data to ensure that diverse populations are adequately represented.

Another important issue is *transparency and explainability*. Many AI systems, especially deep learning models, are often criticized as “black boxes” because their internal decision-making processes are difficult to interpret. In clinical trials, where decisions about recruitment, protocol adjustments, or even interpretation of outcomes can directly affect patient lives, this lack of clarity may undermine confidence in the research. Ensuring accountability means embedding explainability tools into AI models and adopting clear reporting standards that allow stakeholders, including regulators, clinicians, and participants, to understand how conclusions are reached.

*Informed consent* also becomes more complex in trials that use AI. Traditionally, consent focuses on clarifying the goals of the study, potential risks, and benefits. When AI is involved, participants should also be informed about how their data will be used, what role algorithms play in shaping decisions, and what safeguards are in place to protect privacy and prevent misuse. Without this added transparency, participant autonomy may be compromised, weakening the ethical foundation of clinical research.

From a *regulatory perspective*, bodies such as the U.S. Food and Drug Administration (FDA) and the European Medicines Agency (EMA) have taken steps to promote diversity in clinical research and encourage innovative methods to improve representation [12]. However, regulations that specifically address AI in clinical trials remain limited. More detailed guidance is needed to define how fairness audits should be conducted, how algorithmic transparency can be enforced, and how accountability is distributed among developers, sponsors, and regulators. Implementing this framework demands a robust Data Governance policy to establish data ownership and ensure compliance with global regulations like GDPR and HIPAA. It also mandates validation and QC for synthetic data from GANs or SMOTE to avoid introducing spurious clinical correlations.

Finally, the *social context* of AI adoption cannot be ignored. For many historically marginalized communities, trust in clinical trials has been shaped by decades of inequitable practices and, in some cases, unethical research. To overcome this legacy, AI-enabled approaches must be introduced alongside meaningful community engagement. Involving patient advocacy groups, local leaders, and diverse stakeholders in both the design and deployment of these tools is essential to ensure that technologies genuinely reflect the needs and values of the populations they are meant to serve [13].

## Discussion

Artificial intelligence (AI) and machine learning (ML) are emerging as powerful tools for tackling the long-standing issues of equity, diversity, and inclusion (EDI) in clinical research. By identifying patterns of underrepresentation, supporting fairer recruitment processes, and improving the transparency of data analysis, these technologies have the potential to bring clinical trials closer to the realities of the populations they are designed to serve. Yet, it is important to recognize that technological advances alone cannot resolve deep-rooted inequities. Real progress depends on combining AI/ML approaches with policy reforms, meaningful community engagement, and strong ethical safeguards.

One of the most significant challenges lies in bridging the gap between research and practice. Although many proofs of concept studies have shown that fairness aware algorithms can address bias, very few of these approaches have been tested in ongoing randomized clinical trials. Small-scale pilot projects that directly integrate AI-driven recruitment optimization, bias monitoring, and equitable outcome validation into live trials are needed. Such initiatives would provide much-needed evidence on whether these tools are feasible, scalable, and ethically acceptable in real-world clinical environments. Without this step, the role of AI in fostering inclusiveness risks remaining largely theoretical.

Regulatory alignment is another critical factor. While existing guidelines from agencies emphasize the importance of diversity in clinical research, there is limited direction on how AI/ML systems should be evaluated for fairness, explainability, and accountability. Collaborative work among regulators, trial sponsors, technology developers, and patient advocacy groups will be essential to create clear standards. Well-defined guidelines would not only improve transparency and accountability but also strengthen trust and promote wider adoption of AI-based methods.

Community engagement is equally vital. Many historically marginalized populations continue to approach clinical research with caution, shaped by past experiences of exclusion or unethical practices. For AI-driven recruitment strategies to succeed, they must be paired with culturally sensitive outreach, accessible language support, and open communication about how these technologies are being used. Trust cannot be earned through algorithms alone; it requires visible commitment to equity, respect, and patient well-being. For practical adoption, sponsors and CROs must align these frameworks with existing FDA/EMA guidance and GCP standards. Barriers like implementation costs in resource-limited settings should also be addressed to enable equitable global scaling.

Finally, equity in clinical research should be viewed as an evolving goal rather than a fixed achievement. As demographics shift and new disparities come to light, fairness aware AI systems must remain adaptive. This highlights the importance of iterative frameworks in which outcomes from one trial feed back into the design of future studies, creating a cycle of continuous improvement. However, these AI/ML tools face some technical limitations such as regulatory approval, validation across different healthcare systems, and geographical locations.

## Conclusion

This study underscored the persistent inequities that limit both the inclusiveness and the external validity of randomized clinical trials. To address these challenges, we introduced a conceptual AI/ML driven framework designed to embed equity, diversity, and inclusion at every stage of the trial process. The framework emphasizes fairness through pre-trial data assessment, recruitment strategies tailored to diverse populations, ongoing bias detection, and validation of outcomes across demographic groups. By systematically incorporating these elements, the model offers a structured path toward reducing disparities and reinforcing the scientific as well as ethical integrity of clinical research. The success of such a framework, however, cannot rest on technology alone. Effective implementation will require regulatory clarity, transparent reporting practices, and strong partnerships with the very communities that have historically been excluded from research. Future efforts should prioritize pilot studies in active RCTs to examine practical feasibility, scalability, and acceptability in real-world settings. Standardizing fairness metrics, improving explainability in AI models, and fostering collaboration between clinicians, data scientists, ethicists, and policymakers will be key steps toward sustainable adoption.

Looking ahead, expanding empirical validation across therapeutic areas and trial types will help establish the framework’s broader applicability. If implemented responsibly, AI and ML have the potential not only to make trials more representative but also to reshape clinical research into a more inclusive and trusted system. Aligning technological innovation with social and ethical responsibility will allow clinical trials to generate evidence that truly reflects the diversity of the populations they aim to serve. In future, pilot studies with active RCT can be conducted to test practical feasibility and long-term impact on various factors such as participants’ retention, trial outcome, and trust of community can be addressed.

## Data Availability

Not applicable.
